# Expression Profiling of Immune-Related Genes in Different Samples from Patients with Endometriosis Revealed Upregulation of *THBS1*, *CHGB* and *CYR61*

**DOI:** 10.3390/ijms27156894

**Published:** 2026-08-01

**Authors:** Eliana Mihaylova, Dragomira Nikolova, Ralitsa Lechova-Dimitrova, Radoslava Vazharova, Elitza Valerieva, Nadia Magunska, Petia Andreeva, Mihaela Milanova, Ivanka Dimova

**Affiliations:** 1 Department of Biology, Medical Genetics and Microbiology, Faculty of Medicine, Sofia University, 1504 Sofia, Bulgaria; eliandimova@gmail.com (E.M.); radvazh@abv.bg (R.V.); 2 Department of Medical Genetics, Medical University Sofia, 1431 Sofia, Bulgaria; dnikolova@medfac.mu-sofia.bg (D.N.);; 3 SBALAG “Maichin dom”, 2 Zdrave Str., 1431 Sofia, Bulgaria; ralitsa.r.lechova@gmail.com; 4 Medical Complex “Dr. Shterev”, 1330 Sofia, Bulgaria; elitsa.valerieva@gmail.com (E.V.); magunska.n@gmail.com (N.M.); andreivp3@gmail.com (P.A.)

**Keywords:** endometriosis mRNA expression, Nanostring technology, thrombospondin-1, CYR61, CHGB

## Abstract

Endometriosis is a chronic, inflammatory, estrogen-dependent disease that affects approximately 6–10% of women of reproductive age. The present study aimed to investigate the expression of 48 immune-related genes in different types of biological samples from patients with histologically confirmed endometriosis compared to a control group. We analyzed (1) patients’ endometrial tissue, ectopic lesions, and menstrual and venous blood, as well as (2) the control group’s endometrial tissue and menstrual and venous blood. After RNA isolation, expression analysis of 48 immune-related genes was performed using NanoString nCounter^®^ Elements XT technology. All samples were normalized using nSolver Analysis Software, including control normalization and the use of a reference gene, as well as additional approaches for the detection of reliable differential expression. Expressions of the analyzed genes were similar in venous blood between patients and the controls; no significant dysregulation was found in menstrual blood. Most of the analyzed genes were upregulated in patients’ endometria. Three genes attracted our attention due to their overexpression in ectopic lesions. We were able to demonstrate the differential expression of three genes in ectopic lesions, suggesting their association with the development of endometriosis—*THBS1*, *CHGB*, and *CYR61*. The evidently higher expression of *THBS1* in the ectopic lesions nominates this molecule as a potential therapeutic target in endometriosis as well.

## 1. Introduction

Endometriosis is a chronic, inflammatory, estrogen-dependent condition characterized by the presence of endometrial-like tissue outside the uterus. It is most commonly associated with pelvic pain and infertility. This condition results in a wide spectrum of clinical manifestations, from minimal peritoneal lesions to deeply infiltrative disease involving the ovaries, fallopian tubes, rectovaginal septum, bowel, bladder, ureter, and, less commonly, extrapelvic sites such as the pericardium, pleura, and even the brain. It is a complex and still poorly understood condition that significantly impairs the quality of life of affected women and currently has no definitive treatment. The prevalence of pelvic endometriosis in the general female population is estimated to be approximately 6–10% [1,2].

Although the origin and pathogenesis of endometriosis remain unclear, there are multiple theories regarding the mechanism of the disease. Inflammatory processes, heterogeneity in hormone receptors, aromatase activity, and epigenetic modifications have been discussed as potential causes [3]. The genetic–epigenetic theory proposes that endometriosis results from the interaction between inherited genetic susceptibility and epigenetic modifications induced by hormonal and environmental factors [1].

There are different non-invasive diagnostic methods for endometriosis—history taking and physical examination, transvaginal ultrasound, and magnetic resonance imaging (MRI). However, to date, only surgical intervention can definitively confirm the presence of superficial peritoneal endometriosis (approx. 50% of cases) [2].

Currently, there are several potential biomarkers for non-invasive diagnosis, but they have not yet been introduced into clinical practice due to the need for further studies and validation [4].

A large number of transcriptomic studies have been conducted to determine whether gene expression in the endometria of patients with endometriosis differs from that of healthy individuals [5,6]. In this context, studies have identified potential biomarkers that are mainly related to the immune response [7]. Of particular importance, the identification of some of the biomarkers found in stromal cells isolated from menstrual blood strengthens their potential as non-invasive biomarkers [8].

According to Zafari et al. [9], the levels of miRNA 199b-3p, 224-5p, and Let-7d-3p in plasma have the potential to serve as diagnostic biomarkers in patients with endometriosis. These results highlight the importance of peripheral blood as a source of valuable diagnostic information, as well as its possible application in monitoring the effectiveness of therapy [10].

Analysis of the scientific studies published to date highlights the existence of potential biomarkers in both invasively and non-invasively collected samples, and outlines the urgent need to develop non-invasive diagnostic approaches in endometriosis. Many questions related to endometriosis remain unanswered, and the mechanisms underlying the development of the disease are still not well-understood. Due to the lack of effective treatment and late diagnosis of endometriosis, efforts are focused on searching for biomarkers for non-invasive testing and diagnosis of the disease, as well as for its development and treatment.

The present study analyzed the expression of 48 immune-related genes in menstrual and venous blood samples, eutopic endometria, and ectopic lesions from patients with endometriosis. For comparison of the obtained data, menstrual and venous blood samples and healthy endometria from unaffected controls were used. We focused on the identification of differentially expressed genes in endometriosis and the possibility of their application in early detection/monitoring of endometriosis, with the aim to suggest markers for the development of endometriosis as well. The study was designed as a pilot exploratory study aimed at identifying potential candidate biomarkers for further validation in larger cohorts.

## 2. Results

Total RNA expression was evaluated using the nCounter^®^ Elements XT technology (NanoString Technologies), containing 48 gene targets as listed in the Materials and Methods. The panel included six positive controls, six negative controls, and one mRNA reference gene (*ACTB*).

Normalization based on positive controls was performed using the geometric mean, and normalization factors were flagged when they fell outside the acceptable range of 0.3–3.0. Gene content normalization was carried out using the reference gene, also applying geometric mean normalization, with values flagged if normalization factors were outside the 0.1–10.0 range. Background noise was removed by manually excluding mRNAs with counts below the limit of detection (LOD), defined as the mean of negative controls plus two standard deviations. Differential expression was determined by calculating fold changes (FCs) using nSolver ratio outputs derived from normalized count data.

The quality control metric for the limit of detection of the six positive controls assesses whether the signal from the POS_E control probe, introduced at a concentration of 0.5 fM (considered the system’s detection threshold), is significantly higher than signals from the negative control probes. Under acceptable conditions, POS_E counts should exceed background levels. In this study, the POS_E probe signal was higher than that of all negative controls.

In total, 34 genes were expressed above LOD in all 24 samples, with 40 genes in >70% of samples and 44 genes in >50% of samples. The analysis was done only for genes with digital counts in more than 50% of samples per group.

Based on the small number per group (*n* = 3–4), we fully accounted for the biological variability and calculated the standard deviation of digital counts for all analyzed genes in each group. If the variability (SDEV) was less than 30%, we considered a fold change (FC) = 1.5–2 as reliable; FC > 3 in SDEV 30–50% as; FC > 5 in SDEV 50–75% as.

### 2.1. Expression Levels of the Analyzed Genes in Venous Blood (Patients vs. Controls)

Using the nSolver Analysis Software v4.0, three differentially expressed genes from the panel were identified in the patients’ blood samples compared to the blood samples of controls: one overexpressed and two underexpressed genes. Table 1 represents these genes with differential expression (FC > [1.5]), as no one gene showed expression of FC > 2 because blood has higher heterogeneity and a lower signal-to-noise ratio, meaning smaller fold changes are common. The overexpressed gene was *ITM2B* (FC = 1.5; *p* < 0.02). Lower expression was detected for *TRAF4* (FC = −2) and *PSMC4* (FC = −2.5). For four genes (*ASPA*, *CHGB*, *LGI1*, and *MMP2*), we did not perform expression analysis in the venous blood samples, since in most of the samples of this group the expression was below LOD.

### 2.2. Expression Levels of the Analyzed Genes in Menstrual Blood (Patients vs. Controls)

Doing the same analysis in RNA samples from menstrual blood, we detected high biological variability between samples and were unable to detect reliable differential gene expression changes.

### 2.3. Expression Levels of the Analyzed Genes in Endometrium (Patients vs. Controls)

**Figure 1:**
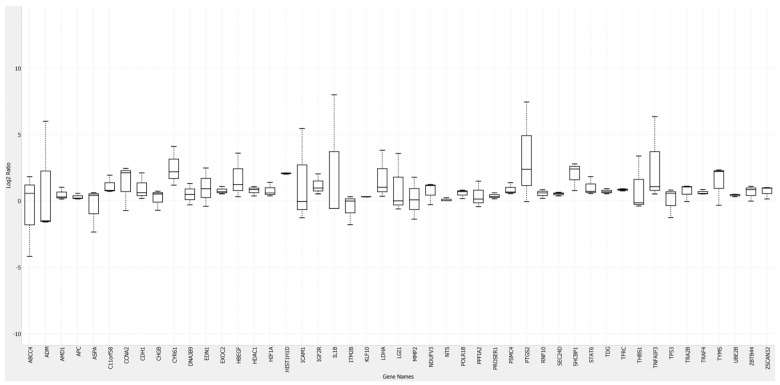
Expression of the analyzed genes in patients’ endometria. Most of the genes showed higher expression in patients’samples (it is above log2 Ratio 0, which corresponds to FC = 1). *CYR61* is the highly overexpressed gene in all patients’ endometria (FC = 5.9 on average).

Most of the analyzed genes showed higher expression in the endometria of patients than in the control endometria, with high biological variability—Figure 1. Markedly higher expression was detected for the gene *CYR61*—FC = 5.9 (*p* < 0.03).

### 2.4. Expression Levels of the Analyzed Genes in Ectopic Lesions

**Figure 2:**
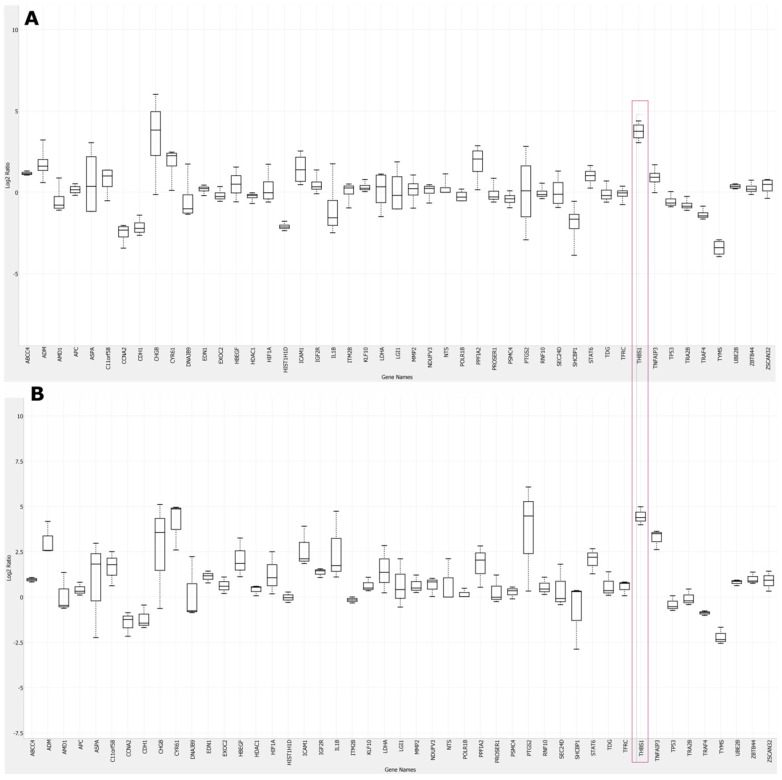
Expression levels of the analyzed genes in ectopic lesions versus patients’ endometria (**A**) and versus the control endometria (**B**). *THBS1* showed markedly elevated expression in both comparisons—showed in red rectangle.

Doing the expression analysis in ectopic lesions, we made two comparisons: (1) with the endometria of patients and (2) with the endometria of controls. Figure 2A represents the expression levels relative to patients’ endometria and Figure 2B to the control endometria.

**Figure 3:**
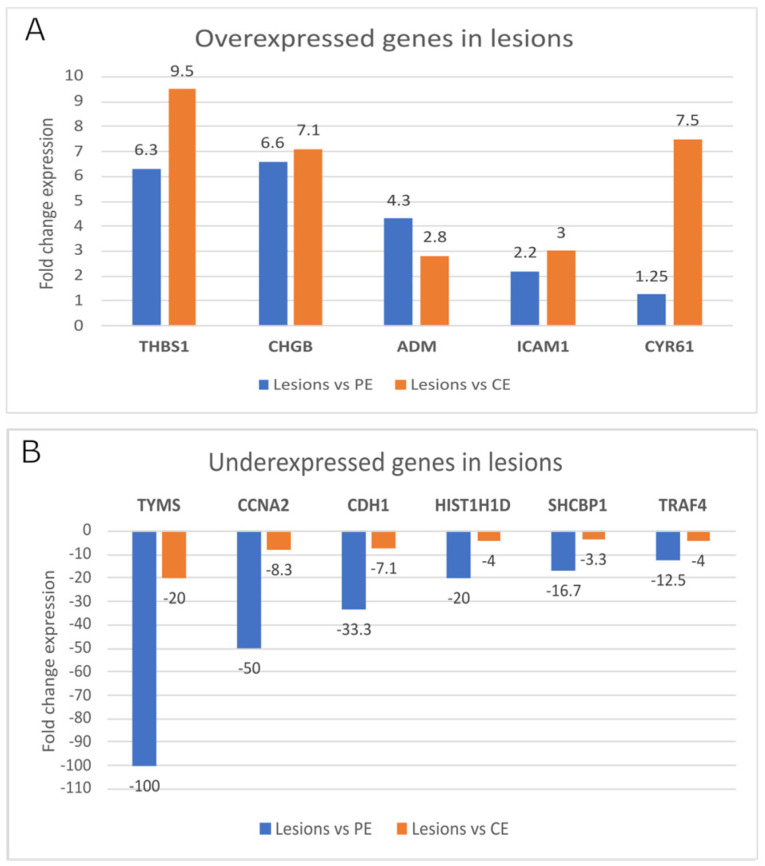
Differentially expressed genes in ectopic lesions compared to patients’ endometria (PE) and control endometria (CE)—(**A**) overexpressed genes and (**B**) underexpressed genes.

The markedly elevated expression levels in both comparisons were detected for the gene *THBS1*—FC = 6.3 vs. patients’ endometria (PE) and FC = 9.5 vs. the control endometria (CE) (*p* < 0.027). Most of the other analyzed genes were underexpressed when compared to patients’ endometria, and only three additional genes were overexpressed without statistical significance: *CHGB* (FC = 6.6), *ADM* (FC = 4.3), and *ICAM1* (FC = 2.2). The same genes were overexpressed when compared to control endometrium as well—*CHGB* (FC = 7.1), *ADM* (FC = 2.8), and *ICAM1* (FC = 3). Their relative expression levels in both comparisons are shown in Figure 3A. The gene *CYR61* was overexpressed (FC = 7.5) when compared to the control endometria (*p* < 0.05), while its FC was 1.25 compared to patients’ endometria.

Six genes were underexpressed in both comparisons (mostly when compared to patients’ endometria): *TYMS* (FC = −100 vs. PE and FC = −20 vs. CE), *CCNA2* (FC = −50 vs. PE and FC = −8.3 vs. CE), *CDH1* (FC = −33.3 vs. PE and FC = −7.1 vs. CE), *HIST1H1D* (FC = −20 vs. PE and FC = −4 vs. CE), *SHCBP1* (FC = −16.7 vs. PE and FC = −3.3 vs. CE), and *TRAF4* (FC = −12.5 vs. PE and FC = −4 vs. CE)—Figure 3B.

Table 2 represents the average fold change expression of the highly overexpressed genes in ectopic lesions.

The most prominent molecule appeared to be *THBS1*, since it had significantly elevated expression in ectopic lesions. Another molecule that was highly expressed in lesions was *CHGB*. *CYR61* showed the highest expression in patients’ endometria and an increase in ectopic lesions as well, when compared to the control endometria.

## 3. Discussion

There is a substantial body of transcriptomic and molecular studies showing altered expression of immune-related genes in endometriosis [11,12,13,14,15]. Altered immune activity has been observed in patients with endometriosis, characterized by elevated levels of inflammatory cytokines and heightened activation of macrophages and neutrophils within the peritoneal cavity [16]. Recently, transcriptome meta-analysis revealed that endometrium immune profile of patients with endometriosis is independent on hormonal milieu [17]. However, only a limited number of studies have compared immune-related gene expression simultaneously in eutopic endometrium, ectopic lesions, menstrual blood, and peripheral blood. This prompted us to investigate immune-related genes with additional cell regulatory functions in different sample types from patients with endometriosis, aiming to find reliable molecules which could serve as biomarkers for the disease.

Doing multiplex mRNA expression analysis by Nanostring technology, we were able to demonstrate differentially expressed immune-related genes in endometria and ectopic lesions from patients with endometriosis. Nanostring is a very powerful technology for the detection of mRNA expression levels, even in low-quantity and low-quality samples. Most of the analyzed genes were upregulated in patients’ endometria compared to the control endometria—a fact that demonstrated the overall high inflammatory state of the endometria of patients with endometriosis. Regarding ectopic lesions, most of the genes were downregulated when compared to patients’ endometria, indicating decreased active inflammation in the lesions. Three genes attracted our attention due to their reliable overexpression in ectopic lesions, which seem to be related to the development of endometriosis—*THBS1*, *CHGB* and *CYR61*.

THBS1 (thrombospondin-1), a multifunctional adhesive glycoprotein that regulates cell-to-cell and cell-to-matrix interactions, is produced and released by multiple cell types [18]—endothelial cells, fibroblasts, and smooth muscle cells; it is the most abundant protein in alpha granules of platelets. THBS1 binds to extracellular matrix ligands, such as fibrinogen, fibronectin, some collagens, latent and active TGFβ1 (transforming growth factor-beta-1), and TNFAIP6 [19]. Its expression is induced by TGFβ, vitamin A, progesterone, and retinoids, modulated by EGF and IFN-gamma [20], and influenced by steroid hormones. It was found that its expression is dependent on steroid hormones 17-beta estradiol and progesterone during in vitro cultivation of human endometrial stromal cells [21].

Although THBS1 has been widely investigated in tumor biology, its role in endometriosis remains poorly explored. Only a small number of studies have mentioned THBS1 so far [22,23]. Recently, Zhang et al. [24] identified THBS1 as an endometriosis biomarker through evidence from single-cell and bulk transcriptomic profiling. It was found as a gene enriched in ectopic endometrial tissue. Its increased expression was confirmed by immunohistochemical analysis, and functional experiments demonstrated that THBS1 enhanced endometrial cell proliferation, migration, and invasion. In a subcutaneous xenograft model, silencing *THBS1* by small interfering RNA reduced the growth of ectopic lesions, highlighting its functional significance *in vivo*. These results suggest that *THBS1* plays a key role in regulating endometrial cellular behavior and provides a mechanistic link between its molecular function and the pathophysiology of endometriosis. By activating the TGFβ pathway, it leads to fibrosis, growth, and persisting lesions. THBS1 interacts with receptors such as CD47, a pathway previously reported to be dysregulated in ectopic endometrial stromal cells [25]. Beyond endometriosis, *THBS1* also plays an important role in reproductive physiology, particularly in angiogenesis, tissue remodeling, and implantation processes, highlighting its broader relevance in reproductive disorders [26]. Our findings support the hypothesis that THBS1 may serve as a potential biomarker for the development of endometriosis. We hypothesize that THBS1, by interacting with integrins, collagen, fibronectin, and matrix metalloproteinases, facilitates the adhesion of endometrial cells to the peritoneum, remodeling of the extracellular matrix, and stabilization of newly formed lesions. Looking to data obtained from DNA microarray experiments that compared gene expression in eutopic endometrium to that of ectopic endometrium, Hansen and Eyster [27] found only 10 genes showing differential expression in three or more experiments in human studies. *THBS1* was found among them, with direct links to TGF-β signaling and NF-κB and IL-6 pathways, i.e., to immune and inflammatory pathways.

CHGB (chromogranin B) is a member of the granin family of acidic proteins and predominantly localized in secretory granules of endocrine and neuroendocrine cells, where it plays an important role in hormone storage, processing, and regulated secretion. It is expressed in multiple tissues, including components of the reproductive system, supporting its potential contribution to reproductive biology and endometrial function [28]. Dysregulation of *CHGB* has been associated with multiple pathological conditions, affecting brain, endocrine, and cardiovascular systems [29]. It has been reported to modulate cellular survival pathways, angiogenesis, and tissue remodeling—key processes in both endometrial physiology and endometriosis progression [30]. Although direct studies investigating CHGB in endometriosis remain limited, its involvement in hormone secretion, angiogenesis, and cellular stress responses indicates that CHGB may represent a potential regulator of cellular behavior in endometriosis—supported by our data with its higher expression in ectopic lesions (about seven times). Chromogranin/secretogranin family proteins (including CHGB) are released during inflammatory responses and participate in immune signaling. They are described as having both pro- and anti-inflammatory properties and being modulated in inflammatory diseases [31].

CYR61 (Cysteine-rich angiogenic inducer 61), also known as CCN1 (Cellular Communication Network Factor 1), is a matricellular protein involved in angiogenesis, cell adhesion, proliferation, and extracellular matrix remodeling—processes that are critical for endometrial physiology and the development of endometriosis [32]. It is an estrogen-responsive growth factor, with its highest expression during the proliferative phase, and aids in uterine repair. *CYR61* is a key deregulated gene in endometriosis, and it is overexpressed in hyperplasia and PCOS [33,34]. CYR61 is secreted by various cell types, including endothelial cells, fibroblasts, and epithelial cells, and mediates cellular signaling through integrin receptors and heparan sulfate proteoglycans. These interactions regulate cell migration, proliferation, and vascular formation, highlighting its importance in reproductive tissue remodeling and lesion establishment [35]. Our results showed its highly elevated expression in the endometria of patients with endometriosis (fold change of 5.9), suggesting its deregulation in these patients. In adults, the expression of CCN proteins is related to inflammation and injury repair and has been demonstrated to be a biomarker for breast cancer, ovarian carcinoma, prostate cancer, and rheumatoid arthritis [36,37].

## 4. Materials and Methods

### 4.1. Study Design and Participants

The present study includes the following groups of participants: patients with endometriosis and non-affected women as controls of the same ethnicity and age range. Persons from vulnerable groups were not included in the study. All participants provided written informed consent and completed a modified World Endometriosis Research Foundation (WERF) questionnaire. All patients were classified according to #ENZIAN classification (2021)—they were affected by ovarian endometriosis, stage O2.

The inclusion and exclusion criteria are presented in Table 3.

### 4.2. Sample Collection and Processing

The following biological materials were collected for analysis: (1) venous blood from the control group (*n* = 4); (2) venous blood from the patient group (*n* = 3); (3) endometrial tissue from the control group (*n* = 4); (4) endometrial tissue from the patient group (*n* = 3); (5) samples from ectopic lesions (*n* = 4); (6) menstrual blood from the control group (*n* = 3); and (7) menstrual blood from the patient group (*n* = 3).

- Venous blood collection—Venous blood containing EDTA was collected from each participant, which was immediately processed to total RNA extraction. Blood samples were collected in the preoperative period—during hospitalization or immediately before surgery—in parallel with standard blood tests prescribed during clinical preparation.
- Tissue collection—All tissue materials were placed in sterile dry containers and stored at −80 °C until RNA isolation. Endometrial tissues from the patients with endometriosis were obtained intraoperatively via cannula by the operating surgeon during a planned laparoscopic intervention under general anesthesia. In the control group, endometrial tissues were collected during a planned hysteroscopy performed for infertility without endometrial pathology. Ectopic lesion samples were obtained intraoperatively during a planned laparoscopic intervention for the treatment of endometriosis under sterile operating conditions.
- Menstrual blood collection—Collection was performed on the second day of the menstrual cycle. Menstrual blood was stored until RNA extraction in a tube with DNA/RNA Shield after being collected with a vaginal swab by the participants themselves or through a cannula by the attending physician.

### 4.3. RNA Extraction

Total RNA from blood samples was isolated using RNAzol^®^ RT RNA Isolation Reagent according to the manufacturer’s protocol (Sigma-Aldrich, 3050 Spruce Street, St. Louis, MO, USA). RNA concentration and purity were measured using a NanoDrop spectrophotometer (NanoDrop™ ND-2000, Thermo Fisher Scientific, Waltham, MA, USA).

Tissue samples were pre-homogenized with the Precellys Soft Tissue Homogenizing CK14-7 mL Kit (Bertin Technologies, Montigny-le-Bretonneux, France), followed by RNA isolation with RNAzol^®^ according to the manufacturer’s protocol. All RNA samples were diluted to the same concentration (25 ng/µL) for gene expression analysis. For the analysis, 5 μL of RNA (125 ng) was used as the input.

### 4.4. Expression Analysis

Gene expression analysis was performed using nCounter^®^ Elements™ XT Reagents (NanoString Technologies, Seattle, WA, USA). For each sample, 5 µL of the same concentration RNA were analyzed using the NanoString nCounter System (NanoString Technologies, Seattle, WA, USA), with a custom panel including 48 immune-related genes and one housekeeping gene (Table 4).

The selection of genes was based on the main suggested pathophysiological mechanisms of endometriosis development, with a focus on inflammation and impaired immune function. We have provided the details for the functions of analyzed genes in Table 4—the second column shows their immune function. The third column presents the additional function of these genes, focusing on their role for proliferation, hormonal signaling, epigenetics, and neuronal function. Endometriotic lesions exhibit significantly higher nerve fiber density (up to 14 times higher) compared to the surrounding normal tissue. Neural cells, particularly peripheral nerves, play a critical and active role in the pathophysiology of endometriosis, transforming it from a solely hormone-dependent gynecological condition into a “nerve-centric” or “exoneural” disease. Endometriotic lesions are densely innervated by sensory and sympathetic nerves that interact with immune cells to drive chronic pain, lesion growth, and inflammation—so the genes with neuronal roles were also included.

The NanoString nCounter assay was performed according to the manufacturer’s protocol (www.nanostring.com). The system uses molecular barcodes and single-molecule visualization to detect and quantify hundreds of unique transcripts within a single reaction. Briefly, total RNA was hybridized in solution with Capture and Reporter probes at 65 °C for 20 h. The samples were then arrayed and fixed onto a cassette in the nCounter Prep Station. After fixation and cleaning of excess elements, the cassettes were placed in the nCounter Digital Analyzer (NanoString Technologies, Seattle, WA, USA). The nCounter Digital Analyzer performs data acquisition by capturing magnified images of the fixed fluorescent molecules using a CCD (charge-coupled device) camera directed through a microscope objective.

To minimize inter-group variation and ensure data consistency, all raw data were pre-processed and normalized using nSolver Analysis Software v4.0 (NanoString Technologies, Seattle, WA, USA). This included background correction using six negative controls, normalization with six positive controls, and reference normalization with the reference gene (*ACTB*). In our experiment, *ACTB* was the highly abundant gene with the average digital counts of expression of 36,498 and an average coefficient of variation 33%. To reduce false-positive signals from low-expression transcripts, a strict LOD threshold was applied. The LOD was defined as the average signal of negative control probes plus two standard deviations. Samples’ mRNA with expression levels below this cutoff were excluded from further analysis.

### 4.5. Statistical Analysis and Gene Expression Determination

Differential Expression Analysis: Changes in mRNA expression were quantified using fold change (FC) values calculated from normalized counts in patients versus controls.

Statistical Validation and Quality Control: Assay performance was further validated by confirming that POS_E control signals at the 0.5 fM detection limit exceeded background levels and exhibited expected linear behavior across all experimental runs.

In nSolver Analysis Software, box plots represent the distribution of gene expression values per sample, displaying the median, interquartile range, and variability. Standard deviation (SD) is typically calculated on normalized counts after background correction, positive control normalization, and normalization by the reference gene, so SD reflects biological + residual technical variability. When samples are assigned to groups (control vs. patients), SD is calculated within each group and is used for calculation of coefficient of variation (CV = SD\mean), which is more informative for expression variability.

Each box plot (as presented in Figure 1 and Figure 2) summarizes expression values for a single gene, showing the center lines (corresponding to median expression); boxes (interquartile range, IQR)—middle 50% of values (25th–75th percentile); whiskers—spread of most data (typically up to 1.5 × IQR); and points outside whiskers (potential outliers).

Differential gene expression between patients and controls was assessed for each gene using an independent-samples *t*-test—we used unpaired, two-sided tests for calculating separate raw *p*-value. To account for multiple comparisons across the 48 genes, *p*-values were adjusted using the Benjamini–Hochberg false discovery rate (FDR) procedure. Adjusted *p*-values (FDR) < 0.05 were considered statistically significant.

In order to overcome the biological variability, standard deviation of digital counts for each gene was calculated for each group. For deviations less than 30%, FC = 1.5–2 was considered as a differential expression, while for 30–50%—FC > 3 and for 50–75%—FC > 5.

Overall, this rigorous analytical approach ensures high confidence in the differential expression analysis, supporting reliable identification of endometriosis-specific alterations.

## 5. Conclusions

In our study, we applied the powerful Nanostring technology for a digital count of mRNA molecules, using a panel of 48 immune-related genes, in different sample types from patients with endometriosis. We tried to find molecules with differential expressions in both tissues and blood/menstrual samples in an attempt to suggest a non-invasive biomarker of the disease. The limitation of our study is the small cohort size, but we provide preliminary evidence for the upregulation of *THBS1* and *CHGB* in ectopic lesions and *CYR61* in eutopic endometria of patients, thus justifying further investigation in larger cohorts. Together, these three genes suggest that lesion establishment depends not only on inflammation but also on coordinated changes in the extracellular matrix (THBS1), vascular remodeling (CYR61), and cellular communication (CHGB). The combined evaluation of *THBS1*, *CYR61*, and *CHGB* could provide a multidimensional view of endometriosis biology. While THBS1 and CYR61 have established roles in tissue remodeling and vascular adaptation, the inclusion of CHGB extends investigation toward secretory and neuro-immune mechanisms that remain poorly characterized in endometriosis. The coordinated dysregulation of these genes may therefore represent a novel molecular signature associated with lesion establishment and persistence, warranting further functional investigation.

To further strengthen the reliability and robustness of our results, an independent validation of these mRNAs should be done in future experiments by quantitative RT-PCR, immunohistochemistry, or ELISA. Even though the study is limited by the number of genes analyzed, we were able to demonstrate differential expressions of these three genes. Further improvement of the sampling for menstrual blood should be done, taking into account its cellular heterogeneity and non-uniform quantity. Matching the sampling to the same menstrual phase is the most reliable method for such investigations.

## Figures and Tables

**Table 1:** Differentially expressed genes in venous blood of patients vs. controls.

Gene	Fold Change	*p*-Value
*ITM2B*	1.5	0.016
*TRAF4*	−2	n.s.
*PSMC4*	−2.5	n.s.

**Table 2:** Average fold change expression of the genes of interest in ectopic lesions.

Gene	FC in Lesions	
	vs. PE	vs. CE
*THBS1*	6.3	9.5
*CHGB*	6.6	7.1
*CYR61*	1.25	7.5

**Table 3:** Inclusion and exclusion criteria for the participants.

Inclusion Criteria	Exclusion Criteria
Female gender Age 18–45 Ability to make independent decisions Lack of severe allergic reactions	Adenomyosis in the control group Adnexitis (pelvic inflammatory disease) in the last 6 months Uterine myomatosis Current pregnancy or pregnancy in the last 6 months (including ectopic) Breastfeeding in the last 3 months Hormone therapy in the last 3 months Diagnosed with malignant neoplasm in the last 12 months Presence of cervical intraepithelial neoplasia (CIN) Chemotherapy or radiotherapy in the last year Comorbidities, such as diabetes, polycystic ovaries, glomerulonephritis, autoimmune diseases, and chronic infections Immune deficiency

**Table 4:** List of the analyzed genes and their function.

Gene	Immune Function	Other Function
*ABCC4*	Regulates export of inflammatory mediators (prostaglandins, cAMP) influencing immune signaling	Membrane transporter (drug resistance, cAMP efflux)
*ACTB*	Structural protein essential for immune cell migration, phagocytosis, and synapse formation	Cytoskeletal structure (β-actin)
*ADM*	Anti-inflammatory peptide; modulates macrophage activity and vascular inflammation	Vasodilation, hormone signaling
*AMD1*	Supports polyamine synthesis, important for lymphocyte proliferation	Polyamine biosynthesis
*APC*	Regulates immune homeostasis via Wnt signaling; impacts T-cell differentiation	Tumor suppressor, Wnt signaling
*ASPA*	Affects neuroinflammation via CNS metabolism	Aspartate metabolism (myelin function)
*BTBD15*	Mediates inflammatory signaling pathways, specifically antiviral responses	Protein–protein interaction
*C11orf58*	Cellular stress responses, particularly in immune-mediated vascular dysfunction and inflammatory disease states	Molecular chaperone
*C13orf23*	Promotes extravasation (migration of leukocytes out of blood vessels) and accumulation of immune cells	Chromatin modification
*CCNA2*	Controls proliferation of immune cells (e.g., activated T cells)	Cell-cycle regulation (S/G2 phase)
*CDH1*	Maintains epithelial barriers; regulates immune cell interactions	Cell adhesion (E-cadherin)
*CHGB*	Involved in neuroendocrine-immune signaling	Secretory granule protein
*CYR61*	Promotes inflammation, leukocyte adhesion, and cytokine production	ECM signaling, angiogenesis
*DNAJB9*	ER (endoplasmic reticulum) stress protein; linked to immune activation and autoimmunity	ER stress response (chaperone)
*EDN1*	Modulates inflammation and leukocyte recruitment via vasoconstriction	Vasoconstrictor peptide
*EXOC2*	Vesicle trafficking; supports immune secretion (e.g., cytokines)	Vesicle trafficking (exocyst complex)
*HBEGF*	Promotes macrophage activation and tissue repair responses	Growth factor signaling
*HDAC1*	Epigenetic regulator controlling cytokine gene expression	Chromatin remodeling
*HIF1A*	Central regulator of immune response under hypoxia; enhances glycolysis in immune cells	Hypoxia response transcription factor
*HIST1H1D*	Chromatin structure; indirectly regulates immune gene transcription	Chromatin structure (histone H1)
*ICAM1*	Key adhesion molecule for leukocyte trafficking and immune synapse formation	Cell adhesion, immune response
*IGF2R*	Modulates macrophage function and antigen processing	Growth factor receptor (lysosomal targeting)
*IL1B*	Major pro-inflammatory cytokine; central to innate immunity	Pro-inflammatory cytokine
*ITM2B*	Role in neuroinflammation regulation	Amyloid processing, neuronal function
*KLF10*	Regulates TGF-β signaling; affects T-cell tolerance and inflammation	Transcription regulation (TGF-β signaling)
*LDHA*	Controls metabolic reprogramming in activated immune cells (e.g., macrophages, T cells)	Glycolysis (lactate production)
*LGI1*	Primarily neuronal role	Synaptic function
*MMP2*	Remodels extracellular matrix; facilitates immune cell migration	ECM degradation
*NDUFV3*	Mitochondrial function; indirectly affects immune cell energy metabolism	Mitochondrial respiration
*NTS*	Modulates neuro-immune interactions	Neuropeptide signaling
*POLR1B*	Supports immune cell proliferation	rRNA transcription
*PPFIA2*	Potential role in mediating inflammation	Synaptic scaffolding
*PSMC4*	Proteasome function; essential for antigen processing (MHC I pathway)	Proteasome ATPase
*PTGS2*	COX-2 enzyme; produces prostaglandins driving inflammation	Inflammation (COX-2 enzyme)
*RNF10*	May regulate immune signaling via ubiquitination	Transcription regulation, ubiquitination
*SEC24D*	Protein trafficking; required for secretion of immune mediators	Vesicle transport (ER to Golgi)
*SFRS10*	RNA splicing; regulates immune gene expression	RNA splicing factor
*SHCBP1*	Promotes proliferation of activated immune cells	Cell-cycle signaling
*STAT6*	Key transcription factor for IL-4/IL-13 signaling (Th2 response)	Cytokine signaling (IL-4/IL-13)
*TDG*	DNA repair; maintains genomic integrity in immune cells	DNA repair (base excision)
*TFRC*	Iron uptake; critical for proliferation of immune cells	Iron uptake (transferrin receptor)
*THBS1*	Regulates inflammation, activates TGF-β, modulates macrophages	Cell–matrix interaction, angiogenesis
*TNFAIP3*	Negative regulator of NF-κB; suppresses inflammation (anti-inflammatory)	NF-κB inhibition (anti-inflammatory)
*TP53*	Controls immune responses via apoptosis, cytokine regulation, and tumor immunity	Tumor suppressor (DNA damage response)
*TRAF4*	Modulates TNF receptor signaling and immune pathways	Signal transduction (TNF receptor pathway)
*TYMS*	DNA synthesis; required for immune cell proliferation	DNA synthesis (thymidylate synthase)
*UBE2B*	Ubiquitination; involved in immune signaling and DNA repair	Ubiquitination enzyme
*ZNF434*	Transcriptional regulation of maturation, differentiation, and function of immune cells	Transcription factor

## Data Availability

Detailed mRNA expression data results can be provided upon reasonable request from the corresponding author. The data are not publicly available due to ethical restrictions.
